# Effects of Warming, Phosphorous Deposition, and Both Treatments on the Growth and Physiology of Invasive *Solidago canadensis* and Native *Artemisia argyi*

**DOI:** 10.3390/plants12061370

**Published:** 2023-03-19

**Authors:** Miaomiao Cui, Bin Yang, Guangqian Ren, Haochen Yu, Zhicong Dai, Jian Li, Qiong Ran, Piergiorgio Stevanato, Justin Wan, Daolin Du

**Affiliations:** 1 Institute of Environment and Ecology, Academy of Environmental Health and Ecological Security, School of the Environment and Safety Engineering, Jiangsu University, Zhenjiang 212013, China; 2 Department of Agricultural Engineering, Jiangsu University, Zhenjiang 212013, China; 3 School of Management, Chongqing University of Technology, Chongqing 400050, China; 4 Department of Agronomy, Food, Natural Resources, Animals and Environment, University of Padova, 35122 Padova, Italy; 5 Jiangsu Collaborative Innovation Center of Technology and Material of Water Treatment, Suzhou University of Science and Technology, Suzhou 215009, China

**Keywords:** global warming, phosphorus, Canadian goldenrod, silvery wormwood, growth, photosynthesis

## Abstract

Anthropogenic climate change and species invasion are two major threats to biodiversity, affecting the survival and distribution of many species around the world. Studying the responses of invasive species under climate change can help better understand the ecological and genetic mechanisms of their invasion. However, the effects of warming and phosphorus deposition on the phenotype of native and invasive plants are unknown. To address the problem, we applied warming (+2.03 °C), phosphorus deposition (4 g m^−2^ yr^−1^ NaH_2_PO_4_), and warming × phosphorus deposition to *Solidago canadensis* and *Artemisia argyi* to measure the direct effects of environmental changes on growth and physiology at the seedling stage. Our results reveal that the physiology parameters of *A. argyi* and *S. canadensis* did not change significantly with the external environment. Under phosphorus deposition, *S. canadensis* had higher plant height, root length, and total biomass compared to *A. argyi*. Interestingly, warming has an inhibitory effect on the growth of both *A. argyi* and *S. canadensis*, but overall, the reduction in total biomass for *S. canadensis* (78%) is significantly higher than *A. argyi* (52%). When the two plants are treated with warming combined with phosphorus deposition, the advantage gained by *S. canadensis* from phosphorus deposition is offset by the negative effects of warming. Therefore, under elevated phosphorus, warming has a negative effect on the invasive *S. canadensis* and reduces its growth advantage.

## 1. Introduction

Global change is an issue of great concern all over the world. The main drivers of global change in terrestrial ecosystems include land-use change, anthropogenic disturbance, climate change, and biological exchange (biological invasion) [1]. Biological invasions are serious environmental problems and are accelerating at an unprecedented rate [2,3,4,5]. There is increasing evidence of the impacts of invasions on global biodiversity, ecosystem functioning, species conservation, and even socioeconomic activities [6,7,8,9]. Therefore, many researchers are very interested in finding the key factors for the success and spread of invasive organisms. As a type of biological invasion, plant invasion processes are driven by changes in other environmental factors [10,11]. Due to the inherent characteristics of invasive plants, they often have serious negative impacts on the native vegetation and local environment in invaded areas, such as high waterlogging tolerance [12], higher phenotypic responses [13,14,15], and biotic characteristics (strong competitiveness against native species) [16,17,18]. Plant invasions result from complex interactions between biotic and abiotic environments under global anthropogenic changes [19].

Environmental shifts due to the impacts of climate change are a global-scale problem. Its main drivers include global warming, increased atmospheric CO_2_ content, nitrogen, and phosphorus deposition [20,21,22,23,24]. Plant growth is strongly driven by various climatic factors, of which temperature is one of the most important drivers. Warming temperatures affect the phenology, reproduction, and competition interactions among native and invasive plant species [25,26,27]. Some studies found that climate warming can put native plants in a more favorable position, such as promoting the seed germination of native plants [28] or increasing the resistance of native plants to invaders [29]. A study found that native plants benefit from warming more than invasive plants, and this asymmetry, in effect, may decrease the relative abundance of invasive plants [29]. Another study found that regardless of warming conditions, invasive and native plants were similar in their responses in terms of total biomass, leaf and root areas, biomass allocation, temperature sensitivity, and phenotypic responses [30]. However, a study found the opposite result, where warming was generally more beneficial to invasive plants than native plants [31]. Warming can increase the ability of invasive plants to acquire resources [28]. In addition, the photosynthetic apparatus of invasive plants are highly thermally stable and has an efficient regulatory mechanism in the energy allocation of PSII complexes, which can minimize potential damage from stress and retain greater carbon assimilation capacity [32].

Phosphorus is an important element necessary for plant growth and is a vital component in plant cell structure and energy production [33]. It is also a structural component of DNA and RNA, and a component of ATP (i.e., energy for metabolic processes) [34,35,36]. At the same time, phosphorus can also participate in the assimilation of plant photosynthesis and the phosphorylation of photosynthesis [37,38,39]. In ecosystems, the main sources of phosphorus deposition are agricultural activities, dust transport, and combustion emissions [24]. The global geometric mean of atmospheric phosphorus wet deposition is 0.21 kg ha^−1^ yr^−1^ [40]. Compared to the years 1959–2000, the atmospheric phosphorus deposition in Asia and Europe has been increasing over the past 20 years. The increase in soil phosphorus can promote the invasion of alien species [41]. However, a study found that phosphorus treatment promoted the growth of native *Pterocypsela laciniata*, but not of invasive *S. canadensis* [42]. Elevated phosphorus can interact with other soil components and have effects on plant growth. For example, prolonged exposure to high phosphorus can exacerbate the negative effects of salt stress on the photosynthetic performance of invasive *Arundo donax* [33]. The contrasting results described above may also be due to different habitat conditions or the plant species [31]. However, to the best of our knowledge, the effects of warming and elevated phosphorus on native and invasive plants have not yet been addressed. Thus, our experiments can contribute to the knowledge of how native and invasive species respond to climate change.

Phenotypic changes in plants to abiotic factors are often in response to stress or stochastic change [43]. At the same time, it is also one of the key factors for plants to maintain good physiological functioning (i.e., homeostasis) [44,45]. Many studies assessed the phenotypic responses to environmental changes, including changes to morphological, biomass, and physiological characteristics [46,47,48]. However, current studies mainly include the effects of either temperature or phosphorus on the phenotypic responses of native and invasive plants [49,50,51,52]. Environmental change factors do not exist alone in the natural environment. Most research on plants tests the combined effects of temperature with nitrogen, carbon dioxide, or water [53,54,55,56]. To the best of our knowledge, the combined effects of temperature and phosphorus on plants have not yet been investigated. Phosphorus deposition is a particularly important global change factor that has a strong impact on plant growth [24], so testing the direct effects of warming and phosphorus on native and invasive plants is important for better predicting the future impacts of invasive species.

In this study, we investigated the growth and physiological responses of seedlings of invasive *Solidago canadensis* and native *Artemisia argyi* to warming and phosphorus treatments. We tested the following hypotheses: (1) warming alone can inhibit the growth of both invasive *Solidago canadensis* and native *Artemisia argyi*; (2) phosphorus deposition alone has a positive effect on the growth of both species; (3) phosphorus deposition can reduce the negative effect of warming on the growth of both species.

## 2. Results

### 2.1. Responses of Growth and Physiological Traits to Environmental Changes

**Figure 1:**
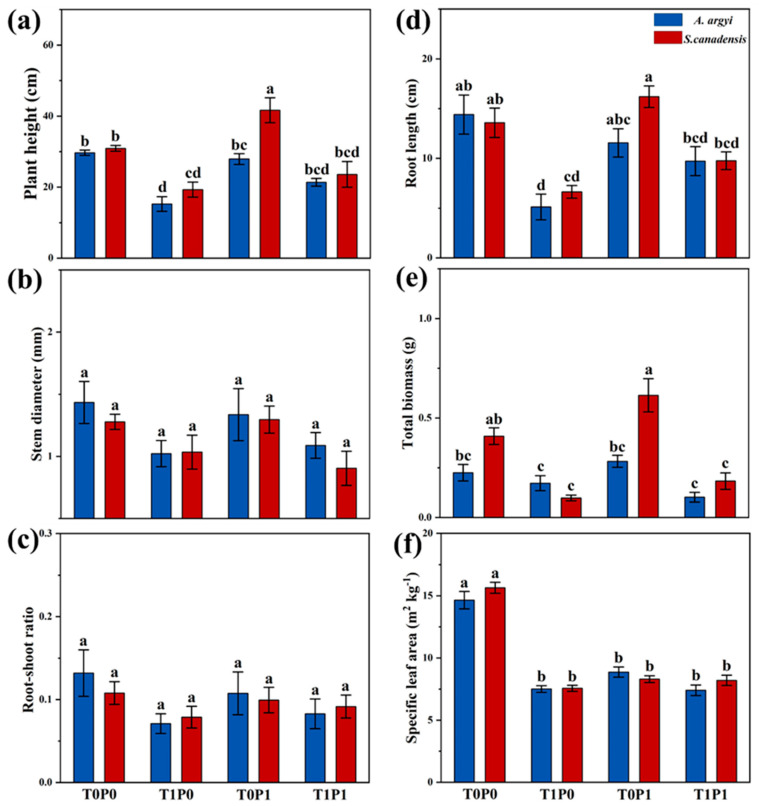
Histograms summarizing the effects of warming, phosphorus deposition, and their interactions on (**a**) plant height, (**b**) stem diameter, (**c**) root–shoot ratio, (**d**) root length, (**e**) total biomass, and (**f**) specific leaf area of native *Artemisia argyi* and invasive *Solidago canadensis*. T0P0: control group, T1P0: warming alone, T0P1: phosphorus deposition alone, and T1P1: warming × phosphorus deposition. The blue boxes represent *A. argyi*, and the red boxes represent *S. canadensis*. Significant differences (*p* < 0.05) between treatments are indicated by different letters. Values are means ± SE.

Warming alone significantly inhibits the growth response parameters of both *S. canadensis* and *A. argyi* (*p* < 0.05, Table 1 and Figure 1), especially plant height, root length, and specific leaf area (*p* < 0.05, Figure 1a,d,f). Plant height, root length, and specific leaf area of *A. argyi* are reduced by 48.6%, 64.4%, and 46.2%, respectively, which is slightly higher than those of *S. canadensis*: 37.5%, 51.1%, and 26.9%, respectively. For plants under phosphorus deposition alone, the total biomass of *S. canadensis* increases significantly by 50.2% compared to the control group, and the final biomass is significantly higher than *A. argyi* (*p* < 0.05, Figure 1e). Under the warming × phosphorus treatment, the specific leaf area of *S. canadensis* and *A. argyi* decreases by 47.6% and 39.7%, respectively, which is significantly lower than the control group (*p* < 0.05, Figure 1f). Correlations among traits are presented in Figure S1.

**Figure 2:**
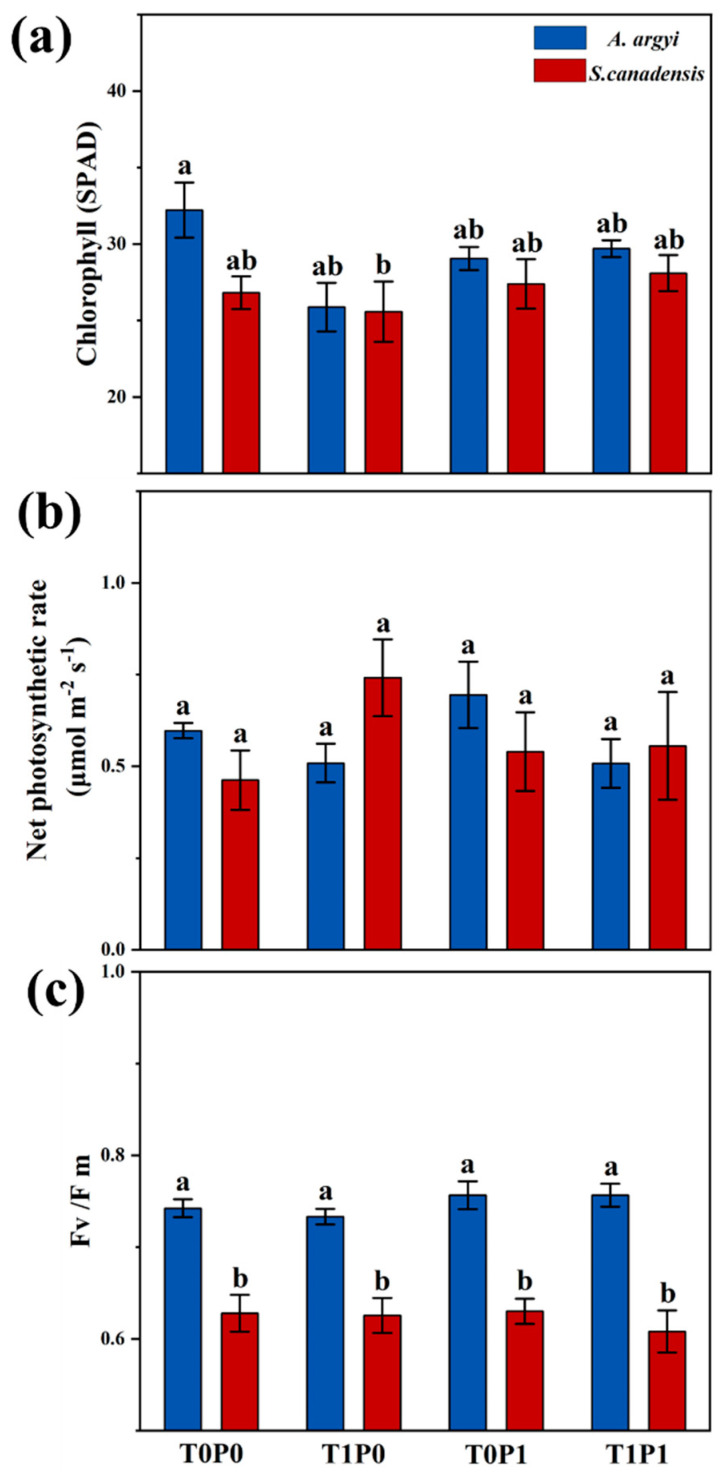
Histograms summarizing the effects of warming, phosphorus deposition, and their interactions on (**a**) chlorophyll, (**b**) net photosynthetic rate, and (**c**) Fv/Fm of native *Artemisia argyi* and invasive *Solidago canadensis*. T0P0: control group, T1P0: warming alone, T0P1: phosphorus deposition alone, and T1P1: warming × phosphorus deposition. The blue boxes represent the *A. argyi*, and the red boxes represent the *S. canadensis*. Significant differences (*p* < 0.05) between treatments are indicated by different letters. Values are means ± SE.

From the three-way ANOVA results, warming alone, phosphorus deposition alone, and warming × phosphorus have no significant effect on net photosynthetic rate and Fv/Fm (i.e., optimal/maximal photochemical efficiency of PS II in the dark, *p* > 0.05, Table 2). The net photosynthetic rate, chlorophyll, and Fv/Fm of *A. argyi* and *S. canadensis* do not show significant changes under any treatment (*p* > 0.05, Figure 2). Interestingly, the Fv/Fm of *S. canadensis* is significantly lower than that of *A. argyi* under all treatments (*p* < 0.05, Figure 2c).

### 2.2. The Effects of Environmental Changes on Plants

**Figure 3:**
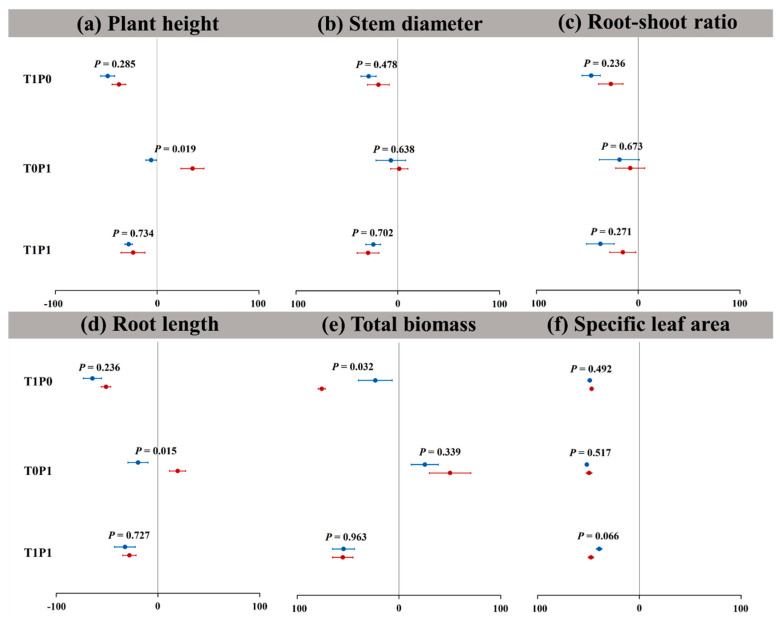
The relative change (%) in (**a**) plant height, (**b**) stem diameter, (**c**) root-shoot ratio, (**d**) root length, (**e**) total biomass, and (**f**) specific leaf area of *Solidago canadensis* and *Artemisia argyi* in response to warming and phosphorus interactions. T1P0: warming alone, T0P1: phosphorus deposition alone, and T1P1: warming × phosphorus deposition. The blue points represent the native *A. argyi*, and the red points represent the invasive *S. canadensis*. *p* < 0.05 means that the relative change rates of the measurement parameters of *A. argyi* and *S. canadensis* have significant differences under the same treatment. Values are mean ± SE (relative changes).

**Figure 4:**
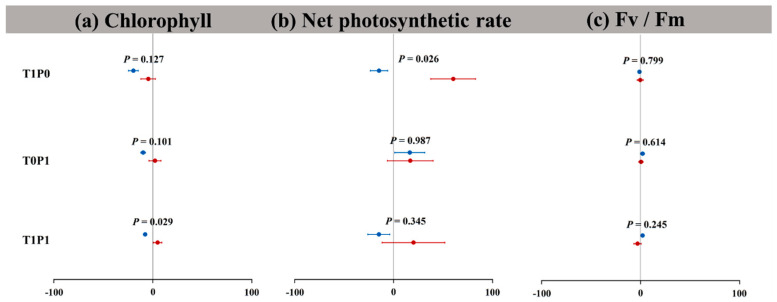
The relative change (%) in (**a**) chlorophyll, (**b**) net photosynthetic rate, and (**c**) Fv/Fm of *Solidago canadensis* and *Artemisia argyi* in response to warming and phosphorus interactions. T1P0: warming alone, T0P1: phosphorus deposition alone, and T1P1: warming × phosphorus deposition. The blue points represent the native *A. argyi*, and the red points represent the invasive *S. canadensis*. *p* < 0.05 means that the relative change rates of the measurement parameters of *A. argyi* and *S. canadensis* have significant differences under the same treatment. Values are mean ± SE (relative changes).

Our results indicate that the relative changes of the two plant growth parameters are negative under warming alone (Figure 3). *S. canadensis* and *A. argyi* have significant differences in biomass parameters (*p* < 0.05, Figure 3e). The relative changes in the net photosynthetic rate of *S. canadensis* and *A. argyi* are, respectively, distributed on both sides of zero, and the relative changes in net photosynthesis rate of the two plants are significantly different (*p* < 0.05, Figure 4). Under phosphorus deposition alone, the relative changes in total biomass, net photosynthetic rate, and Fv/Fm of *A. argyi* are all greater than zero, while the change for other growth indices is variable (Figure 3). For *S. canadensis*, only the relative changes in root–shoot ratio and specific leaf area are less than zero. Under the warming × phosphorus treatment, the relative changes of the two plant growth parameters are less than zero (Figure 3), and there is no significant difference in the relative changes. The relative changes in chlorophyll (less than zero) of *A. argyi* are inversely proportional to those of *S. canadensis* (greater than zero).

## 3. Discussion

Understanding the eco-physiological mechanisms underlying species invasion under contrasting conditions is required for effective management under climate change. Morphological and physiological responses associated with the broad expansion of various invasive species have been described in many empirical studies [48,57,58], while a previous study tested the effects of warming and eutrophication (phosphorus) on the invasive aquatic plant Eichhornia crassipes [59]. To the best of our knowledge, our experiments are the first to address the direct effects of warming and phosphorus deposition on phenotypic changes in native and invasive plants.

### 3.1. Direct Effect of Warming on A. argyi and S. canadensis

Average global temperatures have already risen due to greenhouse gas emissions and are expected to continue rising [60,61]. It is well-established that elevated temperature has a significant impact on plant physiology and growth [62,63,64,65]. Most studies found that invasive plants are generally considered to have a wide range of heat tolerance and high adaptation potential to face new environments [66,67,68]. However, our results did not find that the invasive *S. canadensis* had a stronger adaptation to high temperatures than the native *A. argyi*. Compared with the control group, warming inhibits the growth (i.e., plant height, root length, diameter, total biomass, root-to-shoot ratio, and specific leaf area) of native *A. argyi* and invasive *S. canadensis*. The total biomass reduction rate of *S. canadensis* is significantly higher than that of native *A. argyi*. The net photosynthetic rate and chlorophyll of the two plants does not change significantly under warming. In other words, the ability to accumulate organic matter does not change. However, the net photosynthetic rate is not always directly proportional to the growth response [69]. In low-temperature ecosystems, warming tends to increase plant biomass production because ambient temperatures are often below the optimal growth temperature of plants [70]. In our experiments, all-day warming significantly increased the maximum daily air temperature, likely exposing both plants to heat stress conditions [71]. Therefore, the reduction in the total biomass of the two plant species may be due to shifts in resource allocation, where more resources were consumed to resist the changes in the external environment.

Our results indicate that the growth of both native and invasive plants is inhibited under warming conditions. These results support our first hypothesis that warming alone has inhibitory effects on the growth of both invasive *S. canadensis* and native *A. argyi*. Our experimental results are also similar to previous results finding that warming has a negative effect on plant growth [72,73]. Biomass allocation is often related to plant health and productivity and can vary by species and environmental context [74,75]. Our experimental results show that warming has an inhibitory effect on the total biomass of both plants, but the total biomass of *S. canadensis* has a decreased response to warming. A high root–shoot ratio indicates that the plant has invested heavily in root biomass rather than shoot length and leaf area [76]. The root–shoot ratios of *A. argyi* and *S. canadensis* decrease by 48% and 37%, respectively, but the difference is not significant compared with the control group. Responses in leaf traits are also an important strategy for plants to adapt to their environment. Warming has a significant inhibitory effect on the specific leaf area of both plants. When the specific leaf area is reduced, the resistance of water to the leaf surface increases, which can reduce the water lost by transpiration and put more substances into the construction of strengthening leaf cell walls and vascular structures. This is also a response for protection and to prolong the lifespan of leaves [77,78,79]. In addition, a lower specific leaf area can increase leaf epidermal hair density, mesophyll cell density, and cell wall thickness, thereby achieving the purpose of enhancing leaf stiffness [80].

### 3.2. Direct Effect of Phosphorus on A. argyi and S. canadensis

Compared with the control group, the total biomass of *S. canadensis* and *A. argyi* both respond positively to phosphorus deposition, but at the end, the total biomass of *S. canadensis* is significantly higher than that of *A. argyi*. This may be because the plant height and root length of invasive *S. canadensis* are higher than those of native *A. argyi*. Our second hypothesis that phosphorus deposition alone can promote both *S. canadensis* and *A. argyi* is also partially supported. Longer roots enable greater coverage of soil, reducing spatial heterogeneity, which can promote growth and maintains its competitive advantage [81,82]. In addition, the longer root system provides greater water- and nutrient-foraging capacity for growth promotion [83]. The height advantage can help *S. canadensis* capture light energy more effectively [84,85,86], and increase the distance of seed propagation [57].

Phosphorus treatment does not significantly alter the chlorophyll content, net photosynthetic rate, and Fv/Fm of *S. canadensis* in our experiments. However, the total biomass of *S. canadensis* increases by 50.2%. In our experiments, only the photosynthetic index of leaves was measured, but almost all green organs of plants can carry out photosynthesis, especially young stems or twigs. The rate of photosynthesis in plant stems is usually lower than that in leaves, but the rate can be as high as 75% of leaf photosynthesis [87]. Thus, stem photosynthesis may play an important role in the maintenance of the carbon balance [88]. Thus, increased stem number can also boost net photosynthesis, further enhancing organic matter accumulation.

### 3.3. Direct Effects of Warming and Phosphorus on A. argyi and S. canadensis

A study found that inferring the impact of climate change on plant invasion from signal factors may be misleading [68]. More multifactorial studies are needed to predict plant invasions under global change. As one of the key parameters characterizing invasiveness and adaptability [89], *S. canadensis* investigated in this work exhibited similar biomass to *A. argyi*. Plant responses to phosphorus depend on many factors, including growth conditions such as temperature and light intensity [90], so adding phosphorus cannot fully restore the negative effects of warming on plants. The results also partially support our third hypothesis that phosphorus deposition can reduce the negative effects of warming on *S. canadensis* and *A. argyi*. Some studies found that invasive plants invest more resources in leaf growth (rather than leaf structure per unit area) resulting in higher growth rates [91,92]. We found that the specific leaf area was similar between the two plants, therefore, our results do not reflect those of previous studies. Our results suggest that the dominance of invasive *S. canadensis* under phosphorus deposition can be partially offset by global warming in environments where phosphorus is abundant.

## 4. Material and Methods

### 4.1. Study Species

The experiment was conducted in a greenhouse located at Jiangsu University, Zhenjiang, China. The local climate of the study area is subtropical monsoon, characterized by a mean annual temperature of 16 °C. The annual precipitation is 1297 mm, and the annual hours of sunshine is 1986.9 [93]. In late autumn 2020, seeds of *A. argyi* (native species) and *S. canadensis* (invasive species) were collected from a field in Zhenjiang (119°51′ E, 32°20′ N). In May 2021, seedlings of *A. argyi* and *S. canadensis* were transplanted into monoculture pots (15 cm in diameter and 18 cm in height), each containing four plants. The pots were filled with sand and soil in a 1:1 proportion by volume. The contents of total nitrogen, available phosphorus, and total organ carbon were 3.84 ± 0.20 mg g^−1^, 11.83 ± 0.40 mg kg^−1^, and 6.14 ± 0.12 mg g^−1^, respectively. Throughout the experiment, the ambient average temperature was 28.86 °C, the highest temperature recorded was 34.98 °C, and the lowest was 25.31 °C, and the average humidity was 75.3%. Adequate water was supplied every day during seed germination.

*Solidago canadensis*: A perennial of the Asteraceae family native to North America. It was introduced to China as an ornamental in 1935 [94]. We selected *S. canadensis* for our study for the following reasons. First, *S. canadensis* is among the most notorious weeds. It has invaded Europe, large parts of Asia, Australia, and New Zealand [28,95]. Recently, it was rapidly invading croplands in China and has become very problematic [96]. Second, *S. canadensis* can form monocultures once established, and outcompete native plants in heavily invaded habitats, such as roadsides, abandoned fields, agricultural fields, and pastures [97]. Third, *S. canadensis* is sensitive to climate change and has a strong reproductive capacity (i.e., high seed production and clonal growth) with high ecological adaptability [98];
*Artemisia argyi*: An herbaceous perennial plant with creeping rhizomes also from Asteraceae that can be propagated via seeds and ramets. *A. argyi* is native to China, Korea, Mongolia, and far-eastern Russia [99]. It is distributed broadly across China, except for extremely arid and alpine regions.

### 4.2. Warming and Phosphorus Treatments

**Figure 5:**
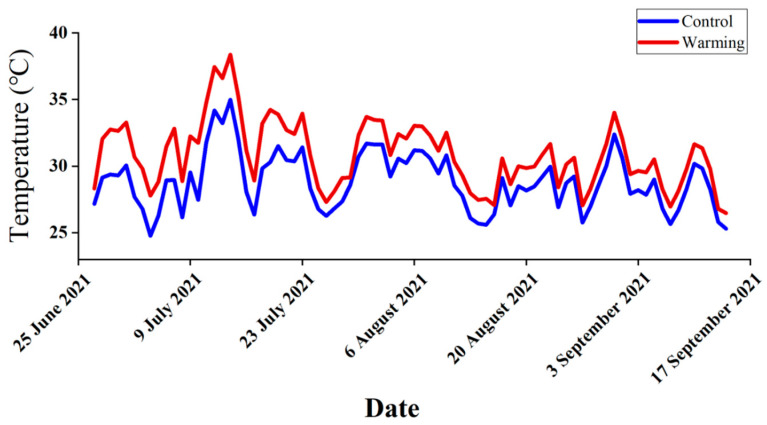
Temperature recorded throughout the experiment. The red line represents the warming treatments, and the blue line represents the control temperature treatments. The experiment ran from 27 June 2021 to 14 September 2021.

Plants were randomly assigned to four treatment groups: (no warming, T0P0), warming alone (all-day warming, T1P0), phosphorus deposition alone (4 g m^−2^ yr^−1^ NaH_2_PO_4_, T0P1), and warming × phosphorus deposition (T1P1). For the warming treatment, a rubber heating plate was used to increase the temperature. The heating plate was placed about 50 cm from the bottom of the flower pot in the warming group [100,101,102], and the heating plate was connected to the temperature controller. The sensors at both ends of the temperature controller were placed in the control and warming treatment groups for temperature control (Figure S1). The temperature and humidity of each group were measured with a temperature and humidity recorder that was placed 5 cm above the plants. Plants within a treatment were shuffled around randomly once a month to minimize greenhouse microclimate effects. During the experiments, the temperature of the warming treatment group was on average 2.03 °C higher than that of the control group (Figure 5). The elevated temperatures simulate global average temperature about 50 years from now [103]. The average deposition of phosphorus was 0.21 ± 0.17 kg P ha^−1^ yr^−1^, and the phosphorus input through wet deposition in China is approximately 0.017g P m^−2^ yr^−1^ [24]. For the phosphorus treatments, NaH_2_PO_4_ solution was added to the soil in pots three times (each time interval is 72 h) before the plants were transplanted into treatments. Plants were watered regularly to prevent water stress. The experiment ran for 90 days in total, from 27 June to 14 September 2021. Each treatment and planting method was replicated five times, involving a total of 40 plants (2 species × 4 treatments × 5 replicates).

### 4.3. Data Collection

In this paper, “biological responses” are the phenotypes of plants, mainly including plant height (Ht), root length (RL), stem diameter (Dt), total biomass (TB), root–shoot ratio (R.S), and specific leaf area (SLA). “Physiological responses” include chlorophyll (Chl), net photosynthetic rate (Pn), and Fv/ Fm. Plant height, root length, and stem diameter were measured using a vernier caliper. The aboveground biomass, underground biomass, and total biomass were harvested, dried, and weighed, to obtain the root–shoot ratio (calculated as underground biomass/aboveground biomass). YMJ-CH intelligent leaf area system (Topu Yunnong Technology, Zhejiang, China) was used to measure leaf area. An electronic balance (BSA124S-CW, Sartorius Scientific Instruments Co., Ltd., Beijing, China) was used to weigh leaves for specific leaf area, which was measured from two fully expanded mature leaves randomly collected from each plant. Photosynthesis is the source of plant energy and can provide the material basis for plants. The photosynthetic process is the result of the interaction of multiple factors, including net photosynthetic rate, chlorophyll, and chlorophyll fluorescence. The changes in photosynthesis traits were assessed to complement the growth data on plant size. All photosynthesis traits were measured between 09:00 and 12:00 on a sunny day, and one random mature leaf from each plant was used for those measurements. We tested the net photosynthetic rate using a plant photosynthesis measurement system (FS-3080H, Fansheng Technology, Hebei, China). A plant nutrition analyzer (TYS-3N, Topu instrument, Zhejiang, China) was used to measure the chlorophyll content of leaves. Fv/Fm was used to gauge whether plants were under stress. Photosynthetic fluorescence (Fv/Fm, a normalized ratio created by dividing variable fluorescence by maximum fluorescence) was measured using Photon Systems Instruments (Czech Republic). The leaves were placed in the dark using a leaf clamp for 15 min before measurement, and the leaves were exposed to the optical probe for the measurement.

### 4.4. Statistical Analyses

Three-way ANOVA was used to assess the statistical differences in the effects of warming (warming and control), phosphorus deposition (elevated and control), and species ID (invasive and native) on plant parameters. Normality and homogeneity were tested by using Shapiro–Wilk test and Levene’s test, respectively. Net photosynthetic rate (Pn) was transformed by natural logarithm to meet the assumptions of ANOVA. One-way ANOVA followed by Tukey post-hoc comparison was performed to compare the effects of different treatments on native *A. argyi* and invasive *S. canadensis*. Two-sample independent *t*-test was also used to examine whether the relative changes in a specific trait differed between native and invasive populations. All statistical analyses were conducted using SPSS Statistics 26 (IBM), and all data were presented in figures using Origin Lab (version Origin 2023 https://www.originlab.com/ (accessed on 29 December 2022)).

To clarify the magnitude of the response of native and invasive plants to different treatments, we also calculated the relative rate of change (RCR) of each parameter:

RCR(%) = [(Trait T − Trait CK)/Trait CK] × 100%(1)

where Trait T represents the index value of the treatment group, and Trait CK represents the index value of the control group. Positive values indicate that environmental changes have a positive effect on species growth, and negative values indicate a negative effect.

## 5. Conclusions

Our results suggest that implementing phosphorus treatment under our experimental conditions promotes the phenotypic responses of *S. canadensis*, which is reflected in the increases in plant height, root length, and biomass parameters. Therefore, the invasion and growth of *S. canadensis* might be accelerated in areas where human agricultural activities intensify (e.g., dust transmission and emissions from combustion sources). However, warming inhibits the growth of *A. argyi* and *S. canadensis* to a certain extent, and the effects on the growth parameters of both plants are consistent. Our findings further validate that warming suppresses the growth of invasive plants [28,29]. In addition, warming could significantly reduce the advantage of phosphorus deposition on invasive *S. canadensis*. In other words, the interaction of the two factors reduced the growth advantage of the invasive *S. canadensis*. This study contributes to understanding the phenotypic responses of *A. argyi* and *S. canadensis* to warming alone, phosphorus deposition alone, and warming × phosphorus deposition. Our results are of great significance for improving the understanding of how invasive and native plants respond to environmental and climate change, including predicting the potential invasion risk of the invasive plant *S. canadensis* under rapid changes in the anthropogenic environment. However, future studies should examine the growth performance of these two species under a wider range of warming and phosphorus deposition, to assess the impact of large-scale environmental changes. These include field transplants that can also further explore these relationships.

## Figures and Tables

**Table 1:** Three-way ANOVA results on the effect of warming (T: control and warming), phosphorus deposition (P: control and elevated), species ID (S: invasive and native), and their interaction effects on plant height, root length, stem diameter, total biomass, root–shoot ratio, and specific leaf area. Statistically significant effects are indicated in bold (* *p* < 0.05, ** *p* < 0.01, *** *p* < 0.001).

Parameter	T	P	S	T × P	T × S	P × S	T × P × S
Plant height	65.96 ***	9.64 **	11.60 **	0.05	1.94	2.93	5.30 *
Root length	42.30 ***	3.96	2.04	4.44 *	0.36	1.12	3.39
Stem diameter	11.40 **	0.14	0.92	0.00	0.00	0.04	0.66
Total biomass	62.19 ***	5.03 *	17.83 ***	4.04	16.96	6.02	0.00
Root–shoot ratio	5.57 *	0.03	0.09	1.22	0.88	0.10	0.08
Specific leaf area	201.76 ***	113.42 ***	1.19	133.72 ***	0.13	0.48	3.83

**Table 2:** Three-way ANOVA results on the effect of warming (T: control and warming), phosphorus deposition (P: control and elevated), species ID (S: native and invasive), and their interaction effects on chlorophyll, net photosynthetic rate, and Fv/Fm. Statistically significant effects are indicated in bold (* *p* < 0.05, *** *p* < 0.001).

Parameters	T	P	S	T × P	T × S	P × S	T × P × S
Chlorophyll	2.49 *	0.90	5.13 *	5.08 *	1.70	0.38	1.62
Net photosynthetic rate	0.01	0.00	0.00	0.97	4.89 *	0.63	0.41
Fv/Fm	0.55	0.25	121.09 ***	0.05	0.11	1.37	0.41

## Data Availability

All raw data will be available upon request.

## Supplementary Materials

The following supporting information can be downloaded at: https://www.mdpi.com/article/10.3390/plants12061370/s1, Figure S1: Correlations between traits in *Solidago canadensis* and *Artemisia argyi*; Figure S2: A schematic diagram of the experimental setup demonstrating the heating equipment and planting methods: (**a**) a representation of the experimental equipment used in the experiment, and (**b**) a schematic diagram of experimental warming and planting methods.

## References

[1] Sala O.E., Chapin F.S., Armesto J.J., Berlow E., Bloomfield J., Dirzo R., Huber-Sanwald E., Huenneke L.F., Jackson R.B., Kinzig A. (2000). Global Biodiversity Scenarios for the Year 2100. Science.

[2] Walther G.-R., Roques A., Hulme P.E., Sykes M.T., Pyšek P., Kühn I., Zobel M., Bacher S., Botta-Dukát Z., Bugmann H. (2009). Alien Species in a Warmer World: Risks and Opportunities. Trends Ecol. Evol..

[3] Wu S., Cheng J., Xu X., Zhang Y., Zhao Y., Li H., Qiang S. (2019). Polyploidy in Invasive *Solidago canadensis* Increased Plant Nitrogen Uptake, and Abundance and Activity of Microbes and Nematodes in Soil. Soil Biol. Biochem..

[4] Shrestha U.B., Shrestha B.B. (2019). Climate Change Amplifies Plant Invasion Hotspots in Nepal. Divers. Distrib..

[5] Robinson T.B., Martin N., Loureiro T.G., Matikinca P., Robertson M.P. (2020). Double Trouble: The Implications of Climate Change for Biological Invasions. NeoBiota.

[6] Dyer E.E., Cassey P., Redding D.W., Collen B., Franks V., Gaston K.J., Jones K.E., Kark S., Orme C.D.L., Blackburn T.M. (2017). The Global Distribution and Drivers of Alien Bird Species Richness. PLoS Biol..

[7] Nguyen N.-A., Eskelson B.N.I., Meitner M.J., Murray T. (2020). People’s Knowledge and Risk Perceptions of Invasive Plants in Metro Vancouver, British Columbia, Canada. Environ. Manag..

[8] Fulgêncio-Lima L.G., Andrade A.F.A., Vilela B., Lima-Júnior D.P., de Souza R.A., Sgarbi L.F., Simião-Ferreira J., De Marco P., Silva D.P. (2021). Invasive Plants in Brazil: Climate Change Effects and Detection of Suitable Areas within Conservation Units. Biol. Invasions.

[9] Wu D., Deng L., Sun Y., Wang R., Zhang L., Wang R., Song Y., Gao Z., Haider H., Wang Y. (2022). Climate Warming, but Not Spartina Alterniflora Invasion, Enhances Wetland Soil HONO and NOx Emissions. Sci. Total Environ..

[10] Deng B., Liu X., Zheng L., Liu Q., Guo X., Zhang L. (2019). Effects of Nitrogen Deposition and UV-B Radiation on Seedling Performance of Chinese Tallow Tree (*Triadica sebifera*): A Photosynthesis Perspective. For. Ecol. Manag..

[11] He Z., He W. (2020). Asymmetric Climate Warming Does Not Benefit Plant Invaders More than Natives. Sci. Total Environ..

[12] Yue M.F., Shen H., Li W.H., Chen J.F., Ye W.H., Tian X.S., Yin A.G., Cheng S.M. (2019). Waterlogging Tolerance of *Bidens Pilosa* Translates to Increased Competitiveness Compared to Native *Bidens biternata*. Plant Soil.

[13] Castillo J.M., Grewell B.J., Pickart A., Bortolus A., Peña C., Figueroa E., Sytsma M. (2014). Phenotypic Plasticity of Invasive *Spartina densiflora* (Poaceae) along a Broad Latitudinal Gradient on the Pacific Coast of North America. Am. J. Bot..

[14] Granata M.U., Bracco F., Catoni R. (2020). Phenotypic Plasticity of Two Invasive Alien Plant Species inside a Deciduous Forest in a Strict Nature Reserve in Italy. J. Sustain. For..

[15] Xiao L., Ding J., Zhang J., Huang W., Siemann E. (2020). Chemical Responses of an Invasive Plant to Herbivory and Abiotic Environments Reveal a Novel Invasion Mechanism. Sci. Total Environ..

[16] Conti L., Block S., Parepa M., Münkemüller T., Thuiller W., Acosta A.T.R., van Kleunen M., Dullinger S., Essl F., Dullinger I. (2018). Functional Trait Differences and Trait Plasticity Mediate Biotic Resistance to Potential Plant Invaders. J. Ecol..

[17] Wang Y.-J., Chen D., Yan R., Yu F.-H., van Kleunen M. (2019). Invasive Alien Clonal Plants Are Competitively Superior over Co-Occurring Native Clonal Plants. Perspect. Plant Ecol. Evol. Syst..

[18] Moura R.F., Queiroga D., Vilela E., Moraes A.P. (2021). Polyploidy and High Environmental Tolerance Increase the Invasive Success of Plants. J. Plant Res..

[19] Liu Y., Sun Y., Müller-Schärer H., Yan R., Zhou Z., Wang Y., Yu F. (2019). Do Invasive Alien Plants Differ from Non-Invasives in Dominance and Nitrogen Uptake in Response to Variation of Abiotic and Biotic Environments under Global Anthropogenic Change?. Sci. Total Environ..

[20] Diffenbaugh N.S., Burke M. (2019). Global Warming Has Increased Global Economic Inequality. Proc. Natl. Acad. Sci. USA.

[21] Yu G., Jia Y., He N., Zhu J., Chen Z., Wang Q., Piao S., Liu X., He H., Guo X. (2019). Stabilization of Atmospheric Nitrogen Deposition in China over the Past Decade. Nat. Geosci..

[22] Hoegh-Guldberg O., Jacob D., Taylor M., Bolaños T.G., Bindi M., Brown S., Camilloni I.A., Diedhiou A., Djalante R., Ebi K. (2019). The Human Imperative of Stabilizing Global Climate Change at 1.5 °C. Science.

[23] Penuelas J., Fernández-Martínez M., Vallicrosa H., Maspons J., Zuccarini P., Carnicer J., Sanders T.G.M., Krüger I., Obersteiner M., Janssens I.A. (2020). Increasing Atmospheric CO_2_ Concentrations Correlate with Declining Nutritional Status of European Forests. Commun. Biol.

[24] Pan Y., Liu B., Cao J., Liu J., Tian S., Du E. (2021). Enhanced Atmospheric Phosphorus Deposition in Asia and Europe in the Past Two Decades. Atmos. Ocean. Sci. Lett..

[25] Cao Y., Xiao Y., Zhang S., Hu W.H. (2018). Simulated Warming Enhances Biological Invasion of *Solidago canadensis* and *Bidens frondosa* by Increasing Reproductive Investment and Altering Flowering Phenology Pattern. Sci. Rep..

[26] Zettlemoyer M.A., Schultheis E.H., Lau J.A. (2019). Phenology in a Warming World: Differences between Native and Non-Native Plant Species. Ecol. Lett..

[27] Su J.Q., Han X.H., Chen B.M. (2021). Do Day and Night Warming Exert Different Effects on Growth and Competitive Interaction between Invasive and Native Plants?. Biol. Invasions.

[28] Zhou X.-H., He W.-M. (2020). Climate Warming Facilitates Seed Germination in Native but Not Invasive Solidago Canadensis Populations. Front. Ecol. Evol..

[29] Wang Z.-X., He Z.-S., He W.-M. (2021). Nighttime Climate Warming Enhances Inhibitory Effects of Atmospheric Nitrogen Deposition on the Success of Invasive *Solidago canadensis*. Clim. Chang..

[30] Zhang X.L., Yu H.H., Lv L., Yang L., Liu C.H., Fan S.F., Yu D. (2021). Effects of Different Scenarios of Temperature Rise and Biological Control Agents on Interactions between Two Noxious Invasive Plants. Divers. Distrib..

[31] Welshofer K.B., Zarnetske P.L., Lany N.K., Read Q.D. (2018). Short-Term Responses to Warming Vary between Native vs. Exotic Species and with Latitude in an Early Successional Plant Community. Oecologia.

[32] Song L., Chow W.S., Sun L., Li C., Peng C. (2010). Acclimation of Photosystem II to High Temperature in Two Wedelia Species from Different Geographical Origins: Implications for Biological Invasions upon Global Warming. J. Exp. Bot..

[33] Cocozza C., Brilli F., Pignattelli S., Pollastri S., Brunetti C., Gonnelli C., Tognetti R., Centritto M., Loreto F. (2020). The Excess of Phosphorus in Soil Reduces Physiological Performances over Time but Enhances Prompt Recovery of Salt-Stressed *Arundo donax* Plants. Plant Physiol. Biochem..

[34] Hawkesford M., Horst W., Kichey T., Lambers H., Schjoerring J., Møller I.S., White P., Marschner P. (2012). Functions of Macronutrients. Marschner’s Mineral Nutrition of Higher Plants.

[35] Roy S., Caruthers M. (2013). Synthesis of DNA/RNA and Their Analogs via Phosphoramidite and H-Phosphonate Chemistries. Molecules.

[36] Scheerer U., Trube N., Netzer F., Rennenberg H., Herschbach C. (2019). ATP as Phosphorus and Nitrogen Source for Nutrient Uptake by *Fagus sylvatica* and Populus x Canescens Roots. Front. Plant Sci..

[37] Ellsworth D.S., Crous K.Y., Lambers H., Cooke J. (2015). Phosphorus Recycling in Photorespiration Maintains High Photosynthetic Capacity in Woody Species. Plant Cell Environ..

[38] Chu S., Li H., Zhang X., Yu K., Chao M., Han S., Zhang D. (2018). Physiological and Proteomics Analyses Reveal Low-Phosphorus Stress Affected the Regulation of Photosynthesis in Soybean. Int. J. Mol. Sci..

[39] Jiang M., Caldararu S., Zaehle S., Ellsworth D.S., Medlyn B.E. (2019). Towards a More Physiological Representation of Vegetation Phosphorus Processes in Land Surface Models. New Phytol..

[40] Zhu J., Wang Q., He N., Smith M.D., Elser J.J., Du J., Yuan G., Yu G., Yu Q. (2016). Imbalanced Atmospheric Nitrogen and Phosphorus Depositions in China: Implications for Nutrient Limitation. J. Geophys. Res. Biogeosci..

[41] King S.A., Buckney R.T. (2002). Invasion of Exotic Plants in Nutrient-Enriched Urban Bushland. Austral Ecol..

[42] Wan L.Y., Qi S.S., Zou C.B., Dai Z.C., Zhu B., Song Y.G., Du D.L. (2018). Phosphorus Addition Reduces the Competitive Ability of the Invasive Weed *Solidago canadensis* under High Nitrogen Conditions. Flora.

[43] Van Kleunen M., Fischer M. (2005). Constraints on the Evolution of Adaptive Phenotypic Plasticity in Plants. New Phytol..

[44] Zhao Y.J., Qing H., Zhao C.J., Zhou C.F., Zhang W.G., Xiao Y., An S.Q. (2010). Phenotypic Plasticity of *Spartina alterniflora* and *Phragmites australis* in Response to Nitrogen Addition and Intraspecific Competition. Hydrobiologia.

[45] Wani G.A., Shah M.A., Tekeu H., Reshi Z.A., Atangana A.R., Khasa D.P. (2020). Phenotypic Variability and Genetic Diversity of *Phragmites australis* in Quebec and Kashmir Reveal Contrasting Population Structure. Plants.

[46] Scheepens J.F., Deng Y., Bossdorf O. (2018). Phenotypic Plasticity in Response to Temperature Fluctuations Is Genetically Variable, and Relates to Climatic Variability of Origin, in Arabidopsis Thaliana. AoB PLANTS.

[47] Wadgymar S.M., Austen E.J. (2019). Shifting Perspectives on the Impacts of Phenotypic Plasticity. New Phytol..

[48] Stotz G.C., Salgado-Luarte C., Escobedo V.M., Valladares F., Gianoli E. (2021). Global Trends in Phenotypic Plasticity of Plants. Ecol. Lett..

[49] Lin Z.H., Wu C.H., Ho C.K. (2018). Warming Neutralizes Host-Specific Competitive Advantages between a Native and Invasive Herbivore. Sci. Rep..

[50] Yu H., Shen N., Yu S., Yu D., Liu C. (2018). Responses of the Native Species Sparganium Angustifolium and the Invasive Species Egeria Densa to Warming and Interspecific Competition. PLoS ONE.

[51] Uddin M.N., Robinson R.W. (2018). Can Nutrient Enrichment Influence the Invasion of *Phragmites australis*?. Sci. Total Environ..

[52] Wang W., Sardans J., Wang C., Zeng C., Tong C., Chen G., Huang J., Pan H., Peguero G., Vallicrosa H. (2019). The Response of Stocks of C, N, and P to Plant Invasion in the Coastal Wetlands of China. Glob. Change Biol..

[53] Hely S.E.L., Roxburgh S.H. (2005). The Interactive Effects of Elevated CO_2_, Temperature and Initial Size on Growth and Competition between a Native C-3 and an Invasive C-3 Grass. Plant Ecol..

[54] Williams A.L., Wills K.E., Janes J.K., Schoor J.K.V., Newton P.C.D., Hovenden M.J. (2007). Warming and Free-Air CO_2_ Enrichment Alter Demographics in Four Co-Occurring Grassland Species. New Phytol..

[55] Blumenthal D.M., Kray J.A., Ortmans W., Ziska L.H., Pendall E. (2016). Cheatgrass Is Favored by Warming but Not CO_2_ Enrichment in a Semi-Arid Grassland. Glob. Change Biol..

[56] Johnson S.N., Hartley S.E. (2018). Elevated Carbon Dioxide and Warming Impact Silicon and Phenolic-Based Defences Differently in Native and Exotic Grasses. Glob. Change Biol..

[57] Zhang R., Jongejans E., Shea K. (2011). Warming Increases the Spread of an Invasive Thistle. PLoS ONE.

[58] Tang L., Wolf A.A., Gao Y., Wang C.H. (2018). Photosynthetic Tolerance to Non-Resource Stress Influences Competition Importance and Intensity in an Invaded Estuary. Ecology.

[59] You W., Yu D., Xie D., Yu L., Xiong W., Han C. (2014). Responses of the Invasive Aquatic Plant Water Hyacinth to Altered Nutrient Levels under Experimental Warming in China. Aquat. Bot..

[60] Rustad L., Campbell J., Marion G., Norby R., Mitchell M., Hartley A., Cornelissen J., Gurevitch J., GCTE-NEWS (2001). A Meta-Analysis of the Response of Soil Respiration, Net Nitrogen Mineralization, and Aboveground Plant Growth to Experimental Ecosystem Warming. Oecologia.

[61] Root T.L., Price J.T., Hall K.R., Schneider S.H., Rosenzweig C., Pounds J.A. (2003). Fingerprints of Global Warming on Wild Animals and Plants. Nature.

[62] Hughes L. (2000). Biological Consequences of Global Warming: Is the Signal Already Apparent?. Trends Ecol. Evol..

[63] Lobell D.B., Field C.B. (2007). Global Scale Climate—Crop Yield Relationships and the Impacts of Recent Warming. Environ. Res. Lett..

[64] Asseng S., Ewert F., Martre P., Rötter R.P., Lobell D.B., Cammarano D., Kimball B.A., Ottman M.J., Wall G.W., White J.W. (2015). Rising Temperatures Reduce Global Wheat Production. Nat. Clim. Change.

[65] Zandalinas S.I., Balfagón D., Gómez-Cadenas A., Mittler R. (2022). Plant Responses to Climate Change: Metabolic Changes under Combined Abiotic Stresses. J. Exp. Bot..

[66] Davidson A.M., Jennions M., Nicotra A.B. (2011). Do Invasive Species Show Higher Phenotypic Plasticity than Native Species and, If so, Is It Adaptive? A Meta-Analysis. Ecol. Lett..

[67] Liu Y., Oduor A.M.O., Zhang Z., Manea A., Tooth I.M., Leishman M.R., Xu X., van Kleunen M. (2017). Do Invasive Alien Plants Benefit More from Global Environmental Change than Native Plants?. Glob. Change Biol..

[68] Yang B., Cui M., Du Y., Ren G., Li J., Wang C., Li G., Dai Z., Rutherford S., Wan J.S.H. (2022). Influence of Multiple Global Change Drivers on Plant Invasion: Additive Effects Are Uncommon. Front. Plant Sci..

[69] Saxe H., Cannell M.G.R., Johnsen Ø., Ryan M.G., Vourlitis G. (2001). Tree and Forest Functioning in Response to Global Warming. New Phytol..

[70] Hudson J.M.G., Henry G.H.R., Cornwell W.K. (2011). Taller and Larger: Shifts in Arctic Tundra Leaf Traits after 16 Years of Experimental Warming. Glob. Change Biol..

[71] Bonamour S., Chevin L.-M., Charmantier A., Teplitsky C. (2019). Phenotypic Plasticity in Response to Climate Change: The Importance of Cue Variation. Philos. Trans. R. Soc. B Biol. Sci..

[72] Jayawardena D.M., Heckathorn S.A., Bista D.R., Mishra S., Boldt J.K., Krause C.R. (2017). Elevated CO_2_ plus Chronic Warming Reduce Nitrogen Uptake and Levels or Activities of Nitrogen-Uptake and -Assimilatory Proteins in Tomato Roots. Physiol. Plant..

[73] Bao X., Wang Z., He Z., He W. (2022). Enhanced Precipitation Offsets Climate Warming Inhibition on *Solidago canadensis* Growth and Sustains Its High Tolerance. Glob. Ecol. Conserv..

[74] Richards C.L., Bossdorf O., Muth N., Gurevitch J. (2006). Jack of All Trades, Master of Some? On the Role of Phenotypic Plasticity in Plant Invasions. Ecol. Lett..

[75] Garbowski M., Avera B., Bertram J.H., Courkamp J.S., Gray J., Hein K.M., Lawrence R., McIntosh M., McClelland S., Post A.K. (2020). Getting to the Root of Restoration: Considering Root Traits for Improved Restoration Outcomes under Drought and Competition. Restor. Ecol..

[76] Chen Q., Hu T., Li X., Song C., Zhu J., Chen L., Zhao Y. (2022). Phosphorylation of SWEET Sucrose Transporters Regulates Plant Root:Shoot Ratio under Drought. Nat. Plants.

[77] Li Y.L., Johnson D.A., Su Y.Z., Cui J.Y., Zhang T.H. (2005). Specific Leaf Area and Leaf Dry Matter Content of Plants Growing in Sand Dunes. Bot. Bull. Acad. Sin..

[78] Wright I.J., Falster D.S., Pickup M., Westoby M. (2006). Cross-Species Patterns in the Coordination between Leaf and Stem Traits, and Their Implications for Plant Hydraulics. Physiol. Plant..

[79] Krober W., Plath I., Heklau H., Bruelheide H. (2015). Relating Stomatal Conductance to Leaf Functional Traits. Jove—J. Vis. Exp..

[80] Coble A.P., Cavaleri M.A. (2015). Light Acclimation Optimizes Leaf Functional Traits despite Height-Related Constraints in a Canopy Shading Experiment. Oecologia.

[81] Davis M.A., Grime J.P., Thompson K., Cui B.J. (2000). Fluctuating Resources in Plant Communities: A General Theory of Invasibility. J. Ecol..

[82] Vaness B.M., Wilson S.D., MacDougall A.S. (2014). Decreased Root Heterogeneity and Increased Root Length Following Grassland Invasion. Funct. Ecol..

[83] Cahill J.F. (2003). Lack of Relationship between Below-Ground Competition and Allocation to Roots in 10 Grassland Species. J. Ecol..

[84] King D.A., Davies S.J., Supardi M.N.N., Tan S. (2005). Tree Growth Is Related to Light Interception and Wood Density in Two Mixed Dipterocarp Forests of Malaysia. Funct. Ecol..

[85] Onoda Y., Saluñga J.B., Akutsu K., Aiba S., Yahara T., Anten N.P.R. (2014). Trade-off between Light Interception Efficiency and Light Use Efficiency: Implications for Species Coexistence in One-Sided Light Competition. J. Ecol..

[86] Pretzsch H. (2014). Canopy Space Filling and Tree Crown Morphology in Mixed-Species Stands Compared with Monocultures. For. Ecol. Manag..

[87] Pfanz H., Aschan G., Langenfeld-Heyser R., Wittmann C., Loose M. (2002). Ecology and Ecophysiology of Tree Stems: Corticular and Wood Photosynthesis. Naturwissenschaften.

[88] Ávila E., Herrera A., Tezara W. (2014). Contribution of Stem CO_2_ Fixation to Whole-Plant Carbon Balance in Nonsucculent Species. Photosynthetica.

[89] Feng Y., Wang J., Sang W. (2007). Biomass Allocation, Morphology and Photosynthesis of Invasive and Noninvasive Exotic Species Grown at Four Irradiance Levels. Acta Oecologica.

[90] Liu D. (2021). Root Developmental Responses to Phosphorus Nutrition. J. Integr. Plant Biol..

[91] Van Kleunen M., Weber E., Fischer M. (2010). A Meta-Analysis of Trait Differences between Invasive and Non-Invasive Plant Species. Ecol. Lett..

[92] Lamarque L.J., Porté A.J., Eymeric C., Lasnier J.-B., Lortie C.J., Delzon S. (2013). A Test for Pre-Adapted Phenotypic Plasticity in the Invasive Tree *Acer negundo* L. PLoS ONE.

[93] Jiang K., Wu B., Wang C., Ran Q. (2019). Ecotoxicological Effects of Metals with Different Concentrations and Types on the Morphological and Physiological Performance of Wheat. Ecotoxicol. Environ. Saf..

[94] Ren G., He F., Sun J., Hu W., Azeem A., Qi S., Yang B., Cui M., Jiang K., Du D. (2021). Resource Conservation Strategy Helps Explain Patterns of Biological Invasion in a Low-N Environment. Biochem. Syst. Ecol..

[95] Ren G., Yang H., Li J., Prabakaran K., Dai Z.C., Wang X.P., Jiang K., Zou C.B., Du D.L. (2020). The Effect of Nitrogen and Temperature Changes on *Solidago canadensis* Phenotypic Plasticity and Fitness. Plant Species Biol..

[96] Wang S., Wei M., Wu B., Cheng H., Wang C. (2020). Combined Nitrogen Deposition and Cd Stress Antagonistically Affect the Allelopathy of Invasive Alien Species Canada Goldenrod on the Cultivated Crop Lettuce. Sci. Hortic..

[97] Czortek P., Królak E., Borkowska L., Bielecka A. (2020). Impacts of Soil Properties and Functional Diversity on the Performance of Invasive Plant Species *Solidago canadensis* L. on Post-Agricultural Wastelands. Sci. Total Environ..

[98] Dong L., Yu H., He W. (2015). What Determines Positive, Neutral and Negative Impacts of *Solidago canadensis* Invasion on Native Plant Species Richness?. Sci. Rep..

[99] Singh B.K., Anupam S. (2011). The Genus Artemisia: A Comprehensive Review. Pharm. Biol..

[100] Yang B., Cui M., Dai Z., Li J., Yu H., Fan X., Rutherford S., Du D. (2022). Non-Addictive Effects of Environmental Factors on Growth and Physiology of Invasive *Solidago canadensis* and a Co-Occurring Native Species (*Artemisia argyi*). Plants.

[101] Zhang T., Tan Y., Zhang H. (2012). Experimental Test on Carbon Crystal Panel System and Simulation Research on Its Partial-Heating Program. Build. Environ..

[102] Benli H., Durmuş A. (2009). Evaluation of Ground-Source Heat Pump Combined Latent Heat Storage System Performance in Greenhouse Heating. Energy Build..

[103] Rogelj J., Meinshausen M., Knutti R. (2012). Global Warming under Old and New Scenarios Using IPCC Climate Sensitivity Range Estimates. Nat. Clim. Chang..

