# Supplementary material for: Effects of Warming, Phosphorous Deposition, and Both Treatments on the Growth and Physiology of Invasive Solidago canadensis and Native Artemisia argyi
Source: Plants (Basel). 2023 Mar 19;12(6):1370. doi: 10.3390/plants12061370 (PMC10051919; doi:10.3390/plants12061370)
Supplement: Supplementary file 1 [file plants-12-01370-s001.zip › plants-2163363-supplementary.pdf]

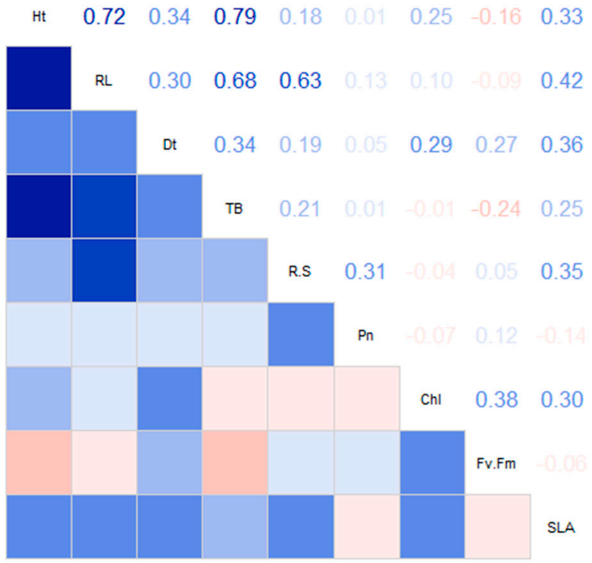

Figure S1. Correlations between traits in *Solidago canadensis* and *Artemisia argyi*.

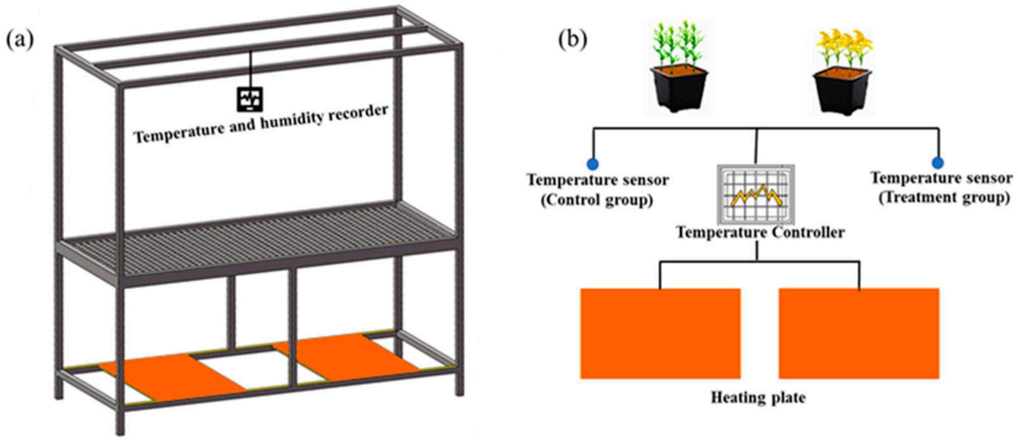

Figure S2. A schematic diagram of the experimental setup demonstrating the heating equipment and planting methods: (**a**) a representation of the experimental equipment used in the experiment, and (**b**) a schematic diagram of experimental warming and planting methods.
